# Supplementary material for: Muscle synergies analysis shows altered neural strategies in women with patellofemoral pain during walking
Source: PLoS One. 2023 Oct 5;18(10):e0292464. doi: 10.1371/journal.pone.0292464 (PMC10553307; doi:10.1371/journal.pone.0292464)
Supplement: Attachment — Submitted filename: Response to Reviewers.docx [file pone.0292464.s002.docx]

September 9^th^, 2023

Emily Chenette

Editors-in-Chief, PLOS ONE

Yaodong Gu

Academic Editor, PLOS ONE

Dear Editors and reviewers,

Attached please find the revised version of our article entitled “**MUSCLE SYNERGIES ANALYSIS SHOWS ALTERED NEURAL STRATEGIES IN WOMEN WITH PATELLOFEMORAL PAIN DURING WALKING**” for possible publication in PLOS One. We truly appreciated the comments from you and the review team.

The manuscript has been revised to fully meet PLOS ONE’s publication criteria. We addressed all points raised by the reviewers and highlighted the new changes in blue in the revised version of the manuscript. We have provided a point-by-point response to each of the reviewer’s comments. We feel that these changes have strengthened the manuscript and thank the reviewers for their helpful comments.

We look forward to hearing your decision about our paper shortly.

Kind regards.

Filipe Oliveira Barroso (on behalf of all co-authors)

**Comments from Editor and reviewers:**

**Editor Comments:**

Please check these minor questions raised by the reviewers.

Authors: We thank the Editor for this comment. All minor questions raised by the reviewers are carefully addressed below. Changes related with each of these questions are highlighted in blue in the revised version of the manuscript.

**Reviewer #1:**

Thank you to the authors for responding to my questions. However, in my opinion, two key points have not been resolved in this revision.
The lack of differences at individual muscle levels needs to be better discussed in the paper. In studies examining neurological diseases (e.g., cerebral palsy or stroke), significant differences have been reported in the number of synergies, VAF, and the pattern of individual muscles. My concern is to give support with example of other studies published where the individual muscle pattern showed no differences, while the muscle synergy analysis did find.

Authors: We thank the reviewer for raising this point again. In the previous revision, we did not understand that the reviewer suggested that if differences in muscle synergies were reported in a given neurological disease, individual muscle EMG differences should (in theory) be also reported.

In addition to the analysis of muscle synergies, differences in individual EMG patterns have been found in some neurological diseases such as stroke (Srivastava et al, 2019; Forman et al, 2019) and cerebral palsy (Cappellini et al, 2023; Forman et al, 2019; Michelsen et al, 2020), for example, but also in PFP (Miao et al, 2015; Bley et al, 2014; Kalytczak et al, 2018). However, these studies mostly focused on hip and knee muscles and compared differences in the activation onset time between pairs of muscles. In our study, we used SPM analysis to look for individual differences in muscle activation between PFPG and CG, but did not find significant differences. In this regard, it is important to highlight that while SPM compares the mean of the linear envelopes of the EMG signals from each subject of each group, the analysis of muscles synergies considers the stride-by-stride variability of each subject and group. Moreover, although we did find differences in the number of muscle synergies between the two groups, no differences were found in terms of recruitment of each synergy throughout the gait cycle (Hs) and the weight of each muscle in each synergy (Ws).

The reduced small sample size, acknowledged as a limitation in the Discussion section, may have affected the results. In fact, although qualitative differences between groups can be observed for some EMGs in Fig 2, these did not reach the significance level of 0.05. The analysis of higher sample sizes could perhaps demonstrate differences in individual muscle patterns when performing SPM analysis.

In any case, when all small individual muscle differences are analyzed as a whole set of muscles with muscle synergies analysis, differences between groups become clearer. This has been explained in lines 393-406 and 443-448 of the revised version of the manuscript.

My second concern is about the lack of association between muscle synergy and clinical variables. Even though this was not the aim of the study, I believe discussing the lack of associations could help suggest that neural strategies might not correlate with clinical symptoms such as pain level or psychological variables (e.g fear of movement, catastrophizing, or depression). Overall, the revised version may be improved if the authors give some space to discuss the above points and contrast the results with the existing literature.

Authors: As suggested by the reviewer, we have included a paragraph discussing the lack of associations between neural strategies and clinical symptoms (lines 413-434).

It is plausible that clinical and psychosocial variables such as catastrophizing, fear of movement and pain would lead individuals with PFP to use different neural strategies to protect against pain during movement. Studies showed an association between kinesiophobia and the reduction of cadence and peak of knee flexion during stair descent (Silva et al, 2019), as well as with peak of internal hip rotation increase during single-leg drop vertical jump (Vasconcelos et al, 2023). In a parallel analysis, which was not in the scope of this study, we found no association between VAF_total_ and clinical variables of pain intensity (NRPS), disability (AKPS), fear of movement (TAMPA), catastrophizing (PCS) and depression (Beck scale). In individuals who suffered an incomplete spinal cord injury, no association was also observed between the number of synergies and the functional scales used to evaluate these patients, indicating that neural strategies for movement performance do not necessarily relate to clinical variables (Pérez-Nombela et al, 2017). Individuals with PFP present high levels of catastrophizing, fear of movement, depression, and anxiety and these variables are associated with pain and disability (Maclachlan et al, 2017). However, catastrophizing and kinesiophobia are not associated with objective function measured in tests such as forward step-down, single leg hop test, and modified star balance test (Priore et al, 2019). In this regard, it is important to highlight that the relationship between biomechanical and clinical variables is still unclear. Therefore, there is a need for future studies with a larger sample size that may better clarify the relationship between neural strategies and clinical and psychosocial variables, which can help achieve better clinical management of patients with PFP.

**Reviewer #2:**

Lines 185-187, the author shall demonstrate the reason for using MDP in this study rather than arbitrarily sum up that it is a better statistical method than others.

Authors: MDP was chosen for unifying and simplifying the understanding of kinematic data by combining all analyzed data into a single curve and calculating the deviation of the movement of the PFPG in relation to the CG throughout the movement cycle (Barton et al, 2012). MDP was applied in previous studies by our group to compare the kinematics of women with PFP with the kinematics of asymptomatic women in various tasks, including gait, proving to be a suitable method for analyzing possible kinematic differences in this population (Lopes Ferreira et al, 2019 and 2020). In addition, the analysis of kinematics through gait indices is a common practice, and among the indices used, the MDP is a more powerful approach than other statistical methods, according to the systematic review presented by Gonçalves SB et al. (2022). We included this information in the text (lines 185-195).
